# Profile and outcomes of acute poisoning in the toxicology treatment and control center at Tanta University Hospital, Egypt

**DOI:** 10.1186/s40360-023-00650-5

**Published:** 2023-02-03

**Authors:** Omar El-Sayed Rageh, Hamdy Khaled Sabra, Abdulrahman Abdullah Alammar, Omar Naif Alanazi, Ayman Nagy, Ibrahim Ali Kabbash

**Affiliations:** 1 Student at Faculty of Medicine, Tanta University, Tanta, Egypt; 2 Forensic Medicine and Clinical Toxicology Department Faculty of Medicine, Tanta University, Tanta, Egypt; 3 Department of Public Health and Community Medicine, Department Faculty of Medicine, Tanta University, Tanta, 31257 Egypt

**Keywords:** Profile, Acute, Poisoning, University Hospital, Egypt

## Abstract

**Background:**

Poisoning is a major health problem especially in developing countries and leads to high morbidity and mortality.

**Aim:**

To identify the profile of acute poisoning in the Toxicology Unit at Tanta University Hospital, Egypt (2017-2021).

**Methods:**

A cross-sectional study using data extracted from medical records from beginning of January 2017 to end of December 2021. Data including demographic data, Glasgow coma scale, type of poisons, manner of poisoning, time of admission and discharge and state at discharge.

**Results:**

This study included 9713 cases. Rodenticides represented the most frequent cause of poisoning among both males (30%) and females (27%). Pharmaceutical drugs, CNS abused pharmaceutical drugs, and chemicals represent the most common categories (24%, 22%, and 21%, respectively) among children (up to 12 years). Rodenticides and pharmaceutical drugs represent the highest categories among other age groups. Evening admissions represented 52% of cases. Glasgow coma scale was 15 among 86.3% of cases. Intentional poisoning was more common than accidental poisoning (58.6% and 34.7%, respectively). One half (52.2%) of the admitted cases were discharged within 24 hours of admission and 44.4% of them were discharged after 48-72 hours. Family request was the main reason of discharge of cases (70.3%), 15.7% were improved, 4% died. Mortality by rodenticide was 12.5%.

**Conclusion:**

Rodenticides, pharmaceutical and CNS abused pharmaceutical drugs were the most common categories of poisoning. Intentional poisoning was more common than accidental poisoning. Rodenticides were responsible for most deaths.

**Supplementary Information:**

The online version contains supplementary material available at 10.1186/s40360-023-00650-5.

## Introduction

Poisons are any substances that can cause harm or death when entering the body. It can be taken by inhalation, ingestion, injection or absorption through the skin. Some substances can cause poisoning immediately with small doses. Others can cause poisoning after long-term exposure to a small or a large dose [1]. Acute poisoning is a major health problem that leads to emergent hospital admission [2, 3]. WHO estimated that, in 2016, unintentional poisoning caused 106,683 deaths and the loss of 6.3 million years of healthy life [3].

Risk factors and causative agents are frequently different according to age groups. In a study about pattern of acute pediatric poisoning in Middle Delta Poison Control Centers among childhood, the most common types of poisoning were corrosive materials, pesticides, and drugs (antipsychotics, anticonvulsants, opioids, antidepressants, and sedative-hypnotics) [4]. In two studies in Egypt, continuous exposure and accessibility of hazards, sociodemographic characteristics, low level of education of mothers, unsafe storage of chemicals, and child behavior were risk factors for unintentional poisoning among children [2, 5].

A study in Romania showed that intentional poisoning is the most common cause of poisoning among teenagers. Females are more vulnerable to suicidal or intentional poisoning than males. Medications, alcohol, and substance abuse are common poisoning agents among teenagers [6]. Studies in Egypt, Turkey and Seri Lanka reported that poisoning in adulthood (above the age of 40) is less frequent than in childhood and teenage. Exposure to chemical substances in adulthood is common with increasing life burdens. It may be intentional, iatrogenic, illegal, or legal therapeutic drugs [7–9].

In a study at tertiary Indian hospital, pesticides, pharmaceutical drugs, and household products were the most common types of acute poisoning. Pesticides were reported as a cause of intentional and accidental poisoning. In agricultural areas, people were poisoned accidentally by pesticides that may be used for suicidal attempts because of their availability. Pharmaceutical drugs are also used for intentional poisoning due to the availability of street drugs and over-the-counter medications. Accidental poisoning by household products were observed in children and the most common products are hydrocarbon and naphthalene [10].

Poison control centers have a wide variety of activities including prevention, recognition, and management of poisoning. Continuous data collection and exploration of the toxicological profile of communities are critical roles of the poison control centers. The changing profile of poisonings needs to be studied on a regional basis as the prevalence and the types of poisoning are different worldwide. Proper reporting of the toxicological trends helps to provide poisoning centers with proper diagnostic and treatment tools [11, 12].

## Objective

This study provided the profile and outcomes of common poisons in the mid-region of the Nile Delta, Egypt (2017-2021). This will help to identify preventive measures to decrease the burden of poisoning.

## Methodology

### Study settings

Tanta University Hospitals are tertiary care health facilities representing the main center of care for the mid-region of the Nile Delta. It serves more than five million citizens. The toxicology Treatment and Control Center affiliated with Tanta University Hospitals diagnoses and treats about 1500-2000 cases annually. It is the main referral center for cases of poisoning in the region of the Nile Delta of Egypt.

### Study design

Single-center cross-sectional study.

### Study sample

We included all admitted cases to the Toxicology Treatment and Control center, Tanta University Hospitals during the period of “January 2017 – December 2021” as a target population; 9713 cases were included.

### Data collection

The authors of the present study utilized the data available in the medical records of the Toxicology Treatment and Control Center, at Tanta University Hospitals. The authors collected data in an extraction excel sheet. These medical records included the following data: demographic data (including age, and gender), Glasgow Coma scale (GCS) (reported during the period of “January 2018- December 2021), data about the state of poisoning as categories (types) of poisons, cause (manner) of poisoning, time of admission and discharge and state at discharge. Cases were diagnosed based on history given by patients and family members, clinical manifestations and investigations if needed.

Authors divided categories of poisons into, rodenticides, pharmaceutical drugs, CNS abused pharmaceutical drugs, chemicals, insecticides, animal envenomation, botulism, and unknown poisons. A list of the common poisons in each category was prepared (supplement file). The researchers of the present study divided age into three age groups: childhood “up to 12” years old, adolescence “13 - <20” years old, early adulthood was “20 - <40” years old, middle age group “40 – 60” years old, and geriatric was “>60” years old. The authors divided the Glasgow coma scale (GCS) into three categories: “≤ 8, 9-14 and 15”. Causes of poisoning were categorized into drug abuse, accidental, intentional (suicidal or homicidal), and not recorded or unknown. The state of discharge was categorized into discharged according to family request (against medical advice), died, discharged under follow-up where the patients is asked to attend follow up visits at hospital to continue treatment, escaped, improved where the patient is not in need of follow up to continue treatment, admitted to intensive care unit (ICU), and not recorded states. The authors divided the time of admission into the morning (8:00 AM - 3:59 PM), evening (4:00 PM - 11:59 PM), and night (12:00 AM - 7:59 AM). Duration of hospital stay was divided into discharged within 24 hours of admission, discharged within 48-72 hours after admission, and discharged > 3 days after admission.

### Statistical analysis

The authors used an excel sheet to collect required data from medical records and IBM SPSS Statistics version 26 to conduct statistical analysis. We estimated the frequency and percentage for qualitative variables and Chi-square for the relations between sociodemographic data and the profile of poisoning. The cutoff point of significance was a *p*-value of ≤ 0.05.

### Ethical consideration

We kept data of the records used in this study strictly confidential. We collected data anonymously and used it only for scientific purpose. The data were extracted from patients’ files after taking permission from the head of the center. The research ethical committee in Faculty of Medicine, Tanta University, gave approval for the study. The committee gave waiver of the consent as data were extracted from files of already discharged patients.

## Results

From 2017 to 2021, the Toxicology Treatment and Control Center received 9713 cases; 44.1% were males and 55.9% were females. Most patients were children, adolescents, and young adults (29.7%, 24.3%, and 36.1%, respectively). We summarized 53 poisons in detail in supplementary tables.

Table 1 shows categories of poisoning in relation to gender. Rodenticides represent the most frequent cause of poisoning (28.7%) followed by CNS abused pharmaceutical drugs (22.2%) and other pharmaceutical drugs (21.3%). Eighty percent of cases of rodenticide poisoning were due to aluminum phosphide and zinc phosphide. Two-thirds of cases of CNS abused pharmaceutical poisoning were due to antidepressants, anti-epileptics, and antipsychotics. Chemical agents and insecticides represented 12.7% and 10.7%, respectively. Among cases of chemical agent poisoning, the main involved substances were corrosives, carbon monoxide, and benzene. Rodenticides, chemicals, insecticides, and animal envenomation were significantly more frequent among males compared to females. Poisoning by pharmaceutical drugs was significantly higher among females (26.3%) compared to males (14.8%).

**Table 1:** Distribution of admitted cases of poisoning in relation to gender and cause

Categories of poisoning	Males		Females		Total		*X*^*2*^	*p*
	n	%	n	%	n	%		
Rodenticides	1047	30.0	1225	27.7	2272	28.7	4.95	0.026
CNS abused drugs	804	23.0	951	21.5	1755	22.2	2.58	0.054
Pharmaceutical drugs	518	14.8	1165	26.3	1683	21.3	153.30	<0.001
Chemicals	511	14.6	490	11.1	1001	12.7	22.10	<0.001
Insecticides	435	12.5	408	9.2	843	10.7	21.17	<0.001
Animal envenomation	35	1.0	22	0.5	57	0.7	6.28	0.012
Botulism	9	0.3	10	0.2	19	0.2	0.01	0.956
Unknown	131	3.8	152	3.4	283	3.4	0.48	0.488
Total	3490	100.0 44.1	4423	100.0 55.9	9713	100.0 100.0	110.00	<0.001

Regarding the age groups**,** pharmaceutical drugs, CNS abused pharmaceutical drugs, and chemicals represented the most frequent poisoning among children (24.7%, 22.6%, and 21.9%, respectively). Rodenticides, pharmaceutical drugs, and CNS abused pharmaceutical drugs were the most frequently encountered poisoning among adolescents (37.3%, 23.3%, and 18.9%, respectively). Again, rodenticides, CNS abused pharmaceutical drugs, and other pharmaceutical drugs represented the highest categories of poisoning among young adults (32.4%, 25.7%, and 19.3%, respectively). Among adults aged 40-60 years, rodenticides (37.3%), CNS abused pharmaceutical drugs (16.7%), and insecticides (16.2%) were the commonest poisoning categories. Among geriatrics ( >60 years), the main cause of poisoning were rodenticides (38.5%) and insecticides (20.8%). Differences in the distribution of poisoning categories in relation to age groups were all statistically significant at *P*<0.001. (Table 2)

**Table 2:** Distribution of admitted cases of poisoning in relation to age in years

Categories of poisoning	Age in years									
	Up to 12		13-<20		20-<40		40-60		>60	
	n	%	n	%	n	%	n	%	n	%
Rodenticides	339	14.4	717	37.3	924	32.4	255	37.3	37	38.5
Pharmaceutical drugs	582	24.7	448	23.3	551	19.3	92	13.5	10	10.4
CNS abused drugs	531	22.6	364	18.9	734	25.7	114	16.7	12	12.5
Chemicals	516	21.9	151	7.9	247	8.6	80	11.7	7	7.3
Insecticides	249	10.6	178	9.3	285	10.0	111	16.2	20	20.8
Animal envenomation	22	0.9	6	0.3	19	0.7	6	0.9	4	4.2
Botulism	2	0.1	5	0.3	7	0.2	5	0.7	0	0.0
Unknown	113	4.8	54	2.6	89	3.1	21	3.1	6	6.3
Total	2354	100	1923	100	2856	100	684	100	96	100
		*29.7*		*24.3*		*36.1*		*8.6*		*1.2*

*p* value for all categories is <0.001

Intentional poisoning was the most frequently represented by 58.6% of all cases followed by accidental poisoning (34.7%). Poisoning by drug abuse represented only 1%. Regarding the categorical causes of poisoning**,** CNS abused pharmaceutical drugs were the most common substances to cause positioning (96.2%). Intentional poisoning was commonly caused by rodenticides (39.3%), CNS abused pharmaceutical drugs (22.3%) and other pharmaceutical drugs (20.4%). Accidental poisoning was usually associated with chemicals (26.5%) and CNS abused pharmaceutical drugs (20.7%), (Table 3)**.**

**Table 3:** Distribution of admitted cases of poisoning in relation to cause of poisoning

Categories of poisoning	Cause of poisoning							
	Drug abuse		Accidental		Intentional		Not recorded/ Unknown	
	n	%	n	%	n	%	n	%
Rodenticides	0	0.0	445	16.2	1821	39.3	6	1.3
Pharmaceutical drugs	1	1.3	470	17.1	946	20.4	266	58.6
CNS abused drugs	75	96.2	567	20.7	1036	22.3	77	17.0
Chemicals	2	2.6	727	26.5	259	5.6	13	2.9
Insecticides	0	0.0	368	13.4	435	9.4	40	8.8
Animal envenomation	0	0.0	18	0.7	0	0.0	39	8.6
Botulism	0	0.0	18	0.7	1	0.01	0	0.0
Unknown	0	0.0	131	4.8	139	3.0	0	0.0
Total	78	100 1.0	2744	100.0 34.7	4637	100.0 58.6	454	100.0 5.7

Concerning the duration of hospital stay of admitted cases, 52.2% of the admitted cases were discharged within 24 hours of admission and 44.4% of cases were discharged after 48-72 hours. Only cases of poisoning by pharmaceutical drugs, CNS abused pharmaceutical drugs, and chemicals tended to stay for a significantly shorter duration. Whereas those suffering from poisoning by rodenticides stayed for a longer duration. Most cases of botulism stayed for only one day (94.7%) as the antidote is not available at university hospitals in Egypt. All cases diagnosed with botulism were referred once diagnosed to the specialized centers of Ministry of health to get the treatment, (Table 4).

**Table 4:** Distribution of admitted cases of poisoning in relation to duration of hospital stay

Categories of poisoning	Duration of hospital stay						*X*^*2*^	*p*
	Discharged same day		2-3 days		>3 days			
	n	%	n	%	n	%		
Rodenticides	986	43.4	1225	53.9	61	2.7	115.3	<0.001
Pharmaceutical drugs	916	54.4	689	40.9	78	4.6	18.94	<0.001
CNS abused drugs	975	55.6	741	42.2	39	2.2	15.51	<0.001
Chemicals	604	60.3	355	35.5	42	4.2	37.85	<0.001
Insecticides	452	53.6	365	43.3	26	3.1	0.79	0.674
Animal envenomation	31	54.4	20	35.1	6	10.5	10.14	0.006
Botulism	18	94.7	0	0.0	1	5.3	15.27	<0.001
Unknown	151	53.4	122	43.1	10	3.5	0.23	0.892
Total	4133	52.2	3517	44.4	263	3.3		

Regarding state at discharge**,** 70.3 % of cases were discharged due to family requests (against medical advice), and 15.7% were discharged after improvement. Overall mortality was at 4.0%. Mortality caused by rodenticides was the highest at 12.5% followed by botulism at 5.3%. (Table 5)

**Table 5:** Distribution of admitted cases of poisoning in relation to state at discharge

Categories of poisoning	Family request		Died		Under follow up		Escaped		Improved		Admitted to ICU	
	n	%	n	%	n	%	n	%	n	%	n	%
Rodenticides	1661	73.1	285	12.5	41	1.8	66	2.9	212	9.3	7	0.3
Pharmaceutical drugs	1187	70.5	6	0.4	99	5.9	64	3.8	327	19.4	0	0.0
CNS abused drugs	1255	71.5	11	0.6	89	5.1	84	4.8	316	18.0	0	0.0
Chemicals	630	62.9	1	0.1	167	16.7	40	4.0	160	16.0	3	0.3
Insecticides	579	68.7	5	0.6	38	4.5	37	4.4	10	17.5	0	0.0
Animal envenomation	38	66.7	1	1.8	8	14.0	0	0.0	184	21.8	0	0.0
Botulism	11	57.9	1	5.3	6	31.6	1	5.3	0	0.0	0	0.0
Unknown	200	70.7	4	1.4	29	10.2	15	5.3	33	11.7	2	0.7
Total	5561	70.3	314	4.0	477	6.0	307	3.9	1242	15.7	12	0.2

Most cases had Glasgow coma scores of 15 (86.3%). A low Glasgow coma score of ≤8 was reported among 10.3% of cases of poisoning by abusing CNS drugs which was the highest among all other categories of poisoning followed by animal and insecticide poisoning (8.0% and 4.2%, respectively). Nearly one-half of cases (52.2%) attended the hospital during the evening shift which was common for all categories of poisoning. Aluminum phosphate rodenticide was by far the most common cause of poisoning as reported among 46.0% of all cases of rodenticide poisoning followed by zinc phosphide (34.0%). Among pharmaceutical drugs, paracetamol, theophylline and oral hypoglycemic drugs were the most frequent (16.7%, 14.6% and 10.6%, respectively). Among CNS abused pharmaceutical drugs, antipsychotics were the most frequent representing 35.8% of all CNS abused pharmaceutical drugs followed by antiepileptics and antidepressants (15.3% and 12.5%, respectively). Among chemical poisoning, 30.2% were due to corrosives and 27.7% were due to carbon monoxide. Organophosphorus insecticides were the most frequently encountered insecticide-causing poisoning (75.1%). (Data in supplementary tables)

## Discussion

Regarding our study population, more than one-half of the cases were females. Similarly, a study conducted in Egypt in Banha Poisoning Center reported that 60.7% of cases were females. Females were more likely to attempt suicide than males using an overdose. Suicidal attempts among females in the Banha study were 55% [13]. In the present study, results showed that intentional poisoning was the most frequent cause of poisoning

This study demonstrated that cases of poisoning by rodenticides were highest among males and females. A study conducted in Eastern Nepal among cases admitted to the intensive care unit (ICU) showed that females represented 62% [14]. Another study was conducted in a teaching hospital in north India to identify the profile of acute poisoning cases, demonstrating that 57% of cases were males [15]. In the present study, rodenticides had the highest frequency between adolescents, young adults, middle age, and geriatrics followed by pharmaceutical drugs (pharmaceutical and CNS abused drugs). In comparison with a study conducted in Malaysia that showed pharmaceutical drugs, pesticides, and household products had the highest frequency among different age groups [16]. There is urgent need to set regulation to restrict availability and use of rodenticides in Egypt.

Our study shows that chemicals, medical and CNS abused pharmaceutical drugs have the highest frequency among children. Similarly, a study conducted in Egypt (Banha Poisoning Center) showed that household chemicals and pharmaceutical drugs were the most common poisons among children (27.8%, and 42.3%, respectively). The curious nature of children to explore strange elements and the simulation of the adults' behaviors of taking pharmaceutical drugs explain why pharmaceutical drugs have the highest frequency among children [17]. Families should receive health education on how to keep pharmaceutical drugs at home away from children’s hand.

Results of the present study revealed that more than one-half and one-third of our population were admitted due to intentional followed by accidental poisoning. Rodenticides were the most common poisons used in intentional poisoning. Similarly, a study conducted in Iran reported that intentional poisoning (51.0%) was more common than accidental poisoning (32.0%) [18]. Similarly, A study conducted in Egypt reported that pesticides and aluminum phosphides (rodenticide) were the most common agents used in suicide attempts [19]. In Jordon, household products (bleaches, detergents, acids, and alkalis) are the most common poisons used in suicidal attempts [20]. Tanta Toxicology Treatment and Control Center mostly serves rural or agricultural areas. This explains why rodenticides were the most common poisons. This was also suggested by a study conducted in India where most cases were from a rural area (55.0%) and aluminum phosphide was the most common poison (50.0%) [21].

In our study population, CNS abused pharmaceutical drugs and chemicals were responsible for most accidental poisoning admissions (47.2%). A study conducted in Saudi Arabia, where accidental poisoning was more common than intentional poisoning (1.3% and 97.8%, respectively), revealed a different cause where the most common poisons were household substances. Chemicals and alcohol sanitizers’ poisonings were the most common among household substances in that study (39.3 % and 17.7 % respectively) [22]. Intentional abuse of pharmaceutical drugs represents only 1% of our population. In comparison with a Chinese study, suicidal exposure was followed by intention (homicidal) exposure (56.7% and 22.1%, respectively) [23].

As regards the duration of hospital stay, most cases were discharged within 24 hours after admission as most cases were discharged according to family request (70.3%). In addition, the majority of admitted cases were alert on admission with a GCS of 15 (86.3%). Cases of botulism were referred to Ministry of Health specialized centers where the antidote are not available at the university hospitals. Families are usually in a hurry to discharge the patients after becoming reassured about their family member’s condition. For milder and more severe cases, families may prefer to be transferred to a private hospital after receiving first aid and stabilization of care to get better accommodation services than that in governmental centers. In contrast, an Indian study reported that the average duration of hospital stay was about 1-4 days and insecticides were responsible for the highest duration of hospital stay (4-12 days) [24].

In the present study, 70.3% were discharged due to family request, 4% died, and 15.7% improved. In a study conducted in Upper Egypt between 2005 and 2010 among cases of Forensic Medicine Laboratory Institute reported a mortality of 53% of confirmed cases. The high mortality in this study could be explained by being all exposed to intentional poisoning whether as an attempt of suicide or homicide [25]. Another Chinese study reported that 93.3% of cases recovered, 1.5% left against medical advice, 1.3% died, and 3.9% were referred to other centers [24]. Rodenticide poisoning showed the highest rate of mortality among other categories (12.5% of rodenticide cases). A systematic review conducted in Iran reported that the mortality rate of aluminum phosphide was 39.6% (95% CI: 31.5% to 47.9%) [26].

## Limitations

One major limitation is the retrospective data collection form patients’ files. The data my show under reporting as some cases may be treated in private centers. Although our study can show the burden of different categories of poisoning in our community, it cannot address risk factors. Other studies should be conducted to assess the risk factors of the increased burden of poisoning and predictors of clinical outcomes of acute poisoning.

## Conclusion

Acute poisoning affects the patients’ lives in multiple ways. Different types of poisons can illicit different outcomes for each patient according to the patient’s own characteristics such as age, gender, and Glasgow coma scale (GCS). The cause or the manner of poisoning is an important factor to limit the search for specific types of poisons and guides toward proper methods of management. Our study provided the profile of poisoning in Tanta University Toxicology unit, Egypt during the four-year period from January 2017-to December 2021.

### Recommendations

We recommend the following:

- Widespread awareness of the harmful effects of common poisons as rodenticides, insecticides, chemicals, and drugs.
- Reinforce widespread awareness of using appropriate protective measures while dealing with insecticides and rodenticides in agricultural regions.
- Support psychologically distressed people to reduce the burden of intentional poisoning.
- There should be regulations restricting use of hazardous rodenticides and to switch from use of aluminum phosphide tablets to zinc phosphide granules which are relatively less toxic.

## Supplementary Information

**Additional file1: Table S1.** Distribution of admitted cases of poisoning in relation to Glasgow coma scale. **Table S2.** Distribution of admitted cases of poisoning in relation to time of admission. **Table S3.** List of common poisons.

## Data Availability

Data are available upon reasonable request from the corresponding author.
