# Supplementary material for: Profile and outcomes of acute poisoning in the toxicology treatment and control center at Tanta University Hospital, Egypt
Source: BMC Pharmacol Toxicol. 2023 Feb 3;24:6. doi: 10.1186/s40360-023-00650-5 (PMC9896829; doi:10.1186/s40360-023-00650-5)
Supplement: Supplementary file 1 — Additional file1: Table S1. Distribution of admitted cases of poisoning in relation to Glasgow coma scale. Table S2. Distribution of admitted cases of poisoning in relation to time of admission. Table S3. List of common poisons. [file 40360_2023_650_MOESM1_ESM.docx]

Table (S1): Distribution of admitted cases of poisoning in relation to Glasgow coma scale

| Categories of poisoning | Glasgow coma scale | | | | | |
| --- | --- | --- | --- | --- | --- | --- |
|  | <8 | | 9-14 | | 15 | |
|  | n | % | n | % | n | % |
| Rodenticides | 52 | 2.6 | 110 | 5.5 | 1821 | 91.8 |
| Medical drugs | 31 | 2.2 | 69 | 5.0 | 1290 | 92.8 |
| CNS drugs | 151 | 10.3 | 318 | 21.6 | 1000 | 68.1 |
| Chemicals | 24 | 2.8 | 46 | 5.4 | 779 | 91.8 |
| Insecticides | 27 | 4.2 | 26 | 4.1 | 585 | 91.7 |
| Animal envenomation | 4 | 8.0 | 1 | 2.0 | 45 | 90.0 |
| Botulism | 0 | 0.0 | 0 | 0.0 | 19 | 100.0 |
| Unknown | 17 | 6.1 | 40 | 14.3 | 223 | 79.6 |
| Total | 306 | 4.6 | 610 | 9.1 | 5762 | 86.3 |

Table (S2): Distribution of admitted cases of poisoning in relation to time of admission

| Categories of poisoning | Time of admission | | | | | | *X^2^* | p |
| --- | --- | --- | --- | --- | --- | --- | --- | --- |
|  | Morning | | Evening | | Night | |  |  |
|  | n | % | n | % | n | % |  |  |
| Rodenticides | 591 | 26.0 | 1295 | 57.0 | 386 | 17.0 | 60.41 | <0.001 |
| Medical drugs | 414 | 24.6 | 839 | 49.9 | 430 | 25.5 | 10.31 | 0.006 |
| CNS drugs | 381 | 21.7 | 869 | 49.5 | 505 | 28.8 | 50.64 | <0.001 |
| Chemicals | 277 | 27.7 | 509 | 50.8 | 215 | 21.5 | 4.09 | 0.130 |
| Insecticides | 240 | 28.5 | 430 | 51.0 | 173 | 20.5 | 6.39 | 0.041 |
| Animal envenomation | 13 | 22.8 | 28 | 49.1 | 16 | 28.1 | 0.96 | 0.617 |
| Botulism | 3 | 15.8 | 6 | 31.6 | 10 | 52.6 | 3.89 | 0.143 |
| Unknown | 69 | 24.4 | 155 | 54.8 | 59 | 20.8 | 0.86 | 0.649 |
| Total | 1988 | 25.1 | 4131 | 52.2 | 1794 | 22.7 | 2788 | <0.001 |

Table (S3): List of common poisons

| Rodenticide: | 2272 |  |
| --- | --- | --- |
| - Aluminum phosphide | 1044 | 46.0 |
| - Carbamate rodenticide | 454 | 20.0 |
| - Zinc phosphide | 774 | 34.0 |
| Pharmaceutical drugs: | 1683 |  |
| - Anticholinergic | 42 | 2.5 |
| - Antihistaminic | 55 | 3.3 |
| - Antihypertensive | 32 | 1.9 |
| - Beta blockers | 74 | 4.4 |
| - Calcium channel blockers | 41 | 2.4 |
| - Cardiac drugs | 69 | 4.1 |
| - Digitalis | 94 | 5.6 |
| - Diuretics | 20 | 1.2 |
| - Insulin | 18 | 1.1 |
| - Iron | 56 | 3.3 |
| - Miscellaneous drugs | 183 | 10.9 |
| - Mixed drugs | 66 | 3.9 |
| - Muscle relaxant | 30 | 1.8 |
| - Non steroid anti-inflammatory | 23 | 1.4 |
| - Oral hypoglycemic | 178 | 10.6 |
| - Paracetamol | 281 | 16.7 |
| - Pregabalin | 26 | 1.5 |
| - Salicylates | 70 | 4.2 |
| - Skeletal muscle relaxant | 20 | 1.2 |
| - Theophylline | 246 | 14.6 |
| - Thyroxine | 27 | 1.6 |
| - Warfarin | 32 | 1.9 |
| CNS abused drugs | 1755 |  |
| - Alcohol | 92 | 5.2 |
| - Antidepressants | 220 | 12.5 |
| - Antiepileptic | 269 | 15.3 |
| - Antipsychotics | 628 | 35.8 |
| - Benzodiazepines | 169 | 9.6 |
| - Cannabis | 93 | 5.3 |
| - CNS depressants | 55 | 3.1 |
| - Heroin | 102 | 5.8 |
| - Night calm | 35 | 2.0 |
| - Sedatives | 22 | 1.3 |
| - Tramadol | 70 | 4.0 |
| Chemical substances | 1001 |  |
| - Benzene | 126 | 12.6 |
| - Carbon monoxide | 277 | 27.7 |
| - Corrosives | 302 | 30.2 |
| - Formalin | 17 | 1.7 |
| - H_2_O_2_ | 12 | 1.2 |
| - Hydrocarbons | 136 | 13.6 |
| - Phenol | 131 | 13.1 |
| Animal envenomation | 57 |  |
| - Snake bite | 18 | 31.6 |
| - Scorpion bite | 1 | 1.8 |
| - Bees bite | 5 | 8.8 |
| - Toxic fish | 33 | 57.8 |
| Insecticides | 843 |  |
| - Different types | 17 | 2.0 |
| - Malathion | 60 | 7.1 |
| - Organophosphorus | 633 | 75.1 |
| - Pesticides | 35 | 4.2 |
| - Pyrethroids | 98 | 11.6 |
| Botulism | 19 | 100.0 |
